# Meeting report and review: Immunological assays and correlates of protection for next‐generation influenza vaccines

**DOI:** 10.1111/irv.12706

**Published:** 2019-12-13

**Authors:** Florian Krammer, Jerry P. Weir, Othmar Engelhardt, Jacqueline M. Katz, Rebecca Jane Cox

**Affiliations:** ^1^ Department of Microbiology Icahn School of Medicine at Mount Sinai New York City NY USA; ^2^ Division of Viral Products, Food and Drug Administration Bethesda MD USA; ^3^ Division of Virology National Institute for Biological Standards and Control Potters Bar UK; ^4^ Formerly Influenza Branch Centers for Disease Control and Prevention (CDC) Atlanta GA USA; ^5^ Department of Clinical Science Influenza Centre University of Bergen Bergen Norway

**Keywords:** CD4, CD8, correlates of protection, HI, influenza, influenza vaccine, MN, mucosal immunity, NI, stalk antibodies

## Abstract

**Background:**

This report summarizes the discussions and conclusions from the “*Immunological Assays and Correlates of Protection for Next‐Generation Influenza Vaccines”* meeting which took place in Siena, Italy, from March 31, 2019, to April 2, 2019.

**Conclusions:**

Furthermore, we review current correlates of protection against influenza virus infection and disease and their usefulness for the development of next generation broadly protective and universal influenza virus vaccines.

## 1 INTRODUCTION

In 1972, Hobson and colleagues published a seminal paper showing that a hemagglutination inhibition (HI) serum antibody titer in the range of 1:18‐1:36 provided 50% protection from influenza A or influenza B virus challenge in adult volunteers.^1^ Of note, many subjects in this study had antibody titers due to natural infection, not vaccination and the challenge strain was partially attenuated.^1^ Since then, the HI titer has been generally adopted as a correlate of protection against influenza virus infection.^2^, ^3^ Importantly, Hobson and colleagues stated in their paper: *“…an unusual finding was that volunteers with no detectable pre‐challenge antibody often seem to be less susceptible to infection than those with pre‐challenge antibody in low titre.*”^1^ Since then, several other immune markers have been reported to correlate with protection, several of them independently of the serum HI antibody titer. These include interferon γ secreting cells, CD8 and CD4 T cells in peripheral blood,^4^, ^5^, ^6^, ^7^, ^8^ neuraminidase (NA) inhibition (NI) antibody titers^9^, ^10^ antibodies measured by single radial hemolysis (SRH),^11^, ^12^ nasal IgA^13^ and, more recently, antibodies that target the hemagglutinin (HA) stalk domain.^14^ In contrast to HI antibodies, which lead to narrow, strain specific protection, these potential new “correlates of protection” are often based on conserved viral epitopes that are also targeted by next‐generation influenza virus vaccines.^15^, ^16^ New immunological assays and novel correlates of protection will be needed to assess immunogenicity and protective efficacy of the next generation of influenza vaccines, including broadly protective universal influenza virus vaccine candidates. This was the central theme of the “Immunological Assays and Correlates of Protection for Next‐Generation Influenza Vaccines” meeting, organized by the International Society for Influenza and other Respiratory Virus Diseases (ISIRV), held in Siena, Italy from March 31, 2019, to April 2, 2019 https://isirv.org/site/index.php/component/content/article/9-events/438-1st-correlates-siena. The meeting was comprised of invited plenary talks, selected oral presentations from submitted abstracts, poster presentations of submitted abstracts, and open discussion periods. The meeting was attended by 220 attendees including scientists from academia, industry, and government public health, standardization and regulatory agencies that develop and evaluate seasonal and pandemic influenza vaccines. It was structured to discuss the value of human challenge studies and cohort studies in defining correlates of protection, the prediction of vaccine performance, correlates of protection in animal models and their translation into humans, the current state of immunological assays and to identify knowledge gaps and chart a path forward for future development of both improved and game changing influenza virus vaccines.

## 2 THE VALUE OF CORRELATES OF PROTECTION FOR VACCINE DEVELOPMENT: TARGET DISCOVERY AND DE‐RISKING OF PRODUCT DEVELOPMENT

According to Plotkin, a correlate of protection is “*responsible for and statistically correlated with protection*.”^17^ An absolute correlate of protection is a threshold of a correlate, for example, a certain titer that is protective. Furthermore, a surrogate marker of protection is an immune marker “*for the true immunologic correlate of protection, which may be unknown or not easily measurable*” but is significantly associated with the true correlate.^17^

Correlates of protection can be obtained by studying natural immunity in animal models or humans (eg, in cohort studies or human challenge experiments). Based on these correlates, vaccine targets can be defined. Vaccines can then be designed to induce the immune response that has been identified as a correlate of protection in a (natural) infection setting. This vaccine‐induced immunity might then also correlate with protection. However, in some cases differences in immunity induced by natural infection versus vaccination against a certain target might exist and the vaccine‐induced protection might not be equal even though the same response is measured. Correlates or surrogates of protection can also be identified in large efficacy trials of vaccines by statistical analysis of immune readouts that correlate with protection.

When a correlate of protection is available, it simplifies vaccine design and facilitates vaccine development. Vaccines that are based on inducing an immune response that correlates with protection have an advantage since a readout exists that can be used for optimization and evaluation of immunogenicity. Furthermore, having a correlate of protection de‐risks vaccine development since one can assess in the pre‐clinical and early clinical phase if the vaccine induces the expected immune response. If an absolute correlate of protection exists, early phase clinical studies will provide an indication if the vaccine will be protective. As an example, developing an HI antibody inducing vaccine allows optimization of the vaccine in pre‐clinical models to induce high titers of HI antibodies. This can be measured readily and success can be determined by the antibody titers reached. If the same vaccine then induces HI titers above a pre‐defined (arbitrary) threshold in early (eg, Phase I) clinical trials, confidence increases that the same vaccine will show protective efficacy in late stage trials.

In addition, influenza virus vaccines have been licensed in some jurisdictions/under some circumstances based on a correlate of protection, whereas in many cases a new vaccine needs to demonstrate clinical efficacy against influenza virus infection/influenza disease. Once a vaccine has obtained licensure, the existence of a correlate of protection makes changes to the product easier, as comparability can be shown with less expensive immunogenicity trials rather than full efficacy trials.

## 3 EXISTING AND NOVEL CORRELATES OF PROTECTION

In addition to the serum HI antibody titer and SRH zone size, several other potential immune correlates of protection have been identified and are the subject of intense ongoing investigations with the goal of improving the ability to predict vaccine performance. Updates on several of these investigations were provided at the meeting (Table 1). Examples of these alternative immune correlates include the serum virus neutralization antibody titer,^18^, ^19^ which often, but not always, aligns with the serum HI antibody titer. In addition, serum neuraminidase inhibition (NI) antibody titers have been identified as independent correlates of protection in field studies by Monto et al and Couch et al.^9^, ^10^, ^20^ Furthermore, this is supported by recent data from an H1N1 human challenge model.^21^ Of note, anti‐HA stalk antibodies can interfere with H6NX‐based NI assays which have to be taken into account in their interpretation.^22^, ^23^ Anti‐NA enzyme‐linked immunosorbent assays (ELISAs), which do not suffer from this shortcoming, have recently been used to show a negative correlation between anti‐NA titers and virus shedding in humans in a cohort study.^24^ In the same cohort, anti‐HA stalk antibodies have been shown to be an independent correlate of protection against both infection as well as symptomatic disease.^14^ Cross reactive CD4+ and CD8+ cells have also been identified as correlates of protection in human challenge and cohort studies.^4^, ^5^, ^6^, ^7^

**Table 1:** Consensus of assays for detection and measurement of influenza correlates of protection

Proposed correlate of protection	Assay for detection of response
Serum hemagglutination inhibition antibodies	HI assay^2^, ^3^
Serum single radial hemolysis antibodies	Single radial hemolysis assay^11^, ^12^
Nasal IgA	Enzyme‐linked immunosorbent assay (ELISA)^13^
Serum virus neutralization antibody titer	Virus neutralization (VN) assay^18^, ^19^
Serum neuraminidase inhibition (NI) antibody titers	Enzyme‐Linked Lectin assay (ELLA)^9^, ^10^
Serum anti‐neuraminidase binding antibody	ELISA^14^, ^24^
HA stalk‐specific antibodies	ELISA^14^
Interferon gamma secreting cells	Interferon gamma Elispot^6^, ^8^
Cross reactive CD4+ cells	Interferon gamma Elispot^5^, ^6^
Cross reactive CD8+ cells	Virus‐specific cytotoxicity, Interferon gamma Elispot^4^, ^6^, ^7^
Antibody‐dependent cellular cytotoxicity (ADCC) Antibody‐dependent cell‐mediated phagocytosis (ADCP)	Antibody‐dependent cellular cytotoxicity (ADCC); antibody‐dependent cell‐mediated phagocytosis (ADCP) reporter and functional assays using specific target proteins^25^, ^26^, ^27^, ^28^
Complement activation	Complement fixation assay
Mucosal antibodies	ELISA
Viral entry inhibition antibodies	Pseudotype particle entry inhibition assays^29^
Antibodies to the ectodomain of the matrix 2 ion channel (M2e), matrix protein 1 (M1) or nucleoprotein (NP)	Cell‐based ELISA or flow cytometry^30^
Influenza virus proteins	Influenza virus protein arrays (IVPM)^31^, ^32^, ^33^, ^34^
Immune markers	Systems immunology^35^, ^36^

Other immunological markers, including antibody effector functions as measured in antibody‐dependent cellular cytotoxicity (ADCC) or antibody‐dependent cell‐mediated phagocytosis (ADCP) assays,^25^, ^26^, ^27^, ^28^ complement activation, mucosal antibody levels, entry inhibition titers as measured by pseudotype particle entry inhibition assays,^29^ antibodies to the ectodomain of the matrix 2 ion channel (M2e), antibodies to matrix protein 1 (M1) and nucleoprotein (NP),^30^ influenza virus protein arrays (IVPM),^31^, ^32^, ^33^, ^34^ and many others are currently being investigated to assess whether they correlate with protection. Importantly, systems immunology approaches are being used to identify new immunological markers that could then be tested for their potential to predict whether protective immunity was induced through vaccination.^35^, ^36^ It is important to note that some immune responses, for example, the priming induced by H5N1 LAIVs, cannot currently be measured with existing assays. However, this priming is clearly the cause of a strong recall response when subjects who received H5N1 LAIV are later boosted with H5N1 IIV.^37^

## 4 IMMUNOLOGICAL ASSAYS: FROM RESEARCH TO CLINICAL TRIALS

A large number of talks during the meeting focused on reviewing existing assays and the development of novel assays for various immune markers and correlates of protection including HI, MN, NI, ADCC, ADCP, anti‐stalk antibody assays, B‐cell assays, T‐cell assays, pseudoparticle entry inhibition assays, and others. The discovery of new immune markers and correlates of protection often starts in academic laboratories where novel assays are developed. While academic laboratories can be highly innovative, less weight is often put on assay qualification, improvement of reproducibility, and reduction of inter‐laboratory variability.

To address these important issues, assay standardization and harmonization were discussed during the meeting. Without standardization, defined thresholds or value ranges for correlates of protection may be questionable due to inter‐laboratory variability. Therefore, it is in the interest of the entire vaccine community to achieve levels of assay standardization that confer confidence in any correlates of protection that may be determined in the future. While the oldest of the used assays, the HI assay, is still variable from laboratory to laboratory, the use of an international biological standard as well as assay protocol harmonization can substantially reduce variability, as demonstrated in international studies coordinated by the Consortium for the Standardization of Influenza Seroepidemiology (CONSISE)^38^, ^39^, ^40^ and others.^41^, ^42^ A highly collaborative approach, involving academic, regulatory, and industrial laboratories, toward standardization and harmonization of HI and VN assays, is ongoing and was described at the meeting^40^ (http://www.flucop.eu/). However, standardizing and harmonizing assays can be difficult and resource intensive, especially for complex methods that measure cell‐mediated immunity or for quantitative B‐cell assays. Some assays, like ELISAs, virus neutralization assays and NI assays may be easier to standardize whether appropriate standards are or become available. 39, 42 For instance, for the assessment of a type of immune response that has not been considered as a correlate in the past, the generation and characterization of an international standard for measuring group 1 stalk antibodies are currently underway as a first step (spearheaded by NIBSC). If proven to be useful, this standard will become available to the scientific community.

For analysis of clinical trials to test vaccines, assays need to be qualified and/or validated according to international regulatory guidelines (eg, International conference on harmonization of technical requirements for registration of pharmaceuticals for human use. ICH harmonized tripartite guideline. Validation of analytical procedures: Text and methodology Q2(R1); EMEA/CHMP/EWP/192217/2009 Rev. 1 Corr. 2 Committee for Medicinal Products for Human Use (CHMP): Guideline on bioanalytical method validation.). Standardization of sampling is also useful in this context. For pivotal clinical trials (Phase III), assays should be fully validated before initiation of testing.

## 5 STUDIES TO DEFINE NOVEL CORRELATES OF PROTECTION

Several types of studies in humans can be used to identify or confirm correlates of protection. During the meeting, the controlled human influenza virus infection model (CHIVIM),^43^, ^44^ cohort studies, and vaccine field studies were discussed.

Discussion of the CHIVIM was especially timely as a follow‐up to a recent meeting devoted to this topic^43^, ^44^ and because of renewed interest in using human challenge studies to understand correlates of protection and potentially predict vaccine performance. The CHIVIM was established in the 1940s; in early studies, subjects were exposed to aerosolized virus but later intranasal instillation was used for increased safety. This model also was used by Hobson and colleagues to establish the HI titer as correlate of protection.^1^

In the CHIVIM, subjects are pre‐screened for low HI titers to the challenge virus before enrollment. The advantage of the model is the controlled environment, the defined challenge dose with a virus of choice and the ability to measure a multitude of readouts including respiration, heart rate, blood pressure, and other cardiac measures as well as the ability to collect multiple biological samples (for instance, from the upper and lower respiratory tract) which allows for in depth immunological analysis. The disadvantages of the model are that it does not closely resemble natural infection since very large challenge virus doses must be used, that it only induces mild upper respiratory tract infections, that it can only be used in healthy adults and not in influenza risk groups (eg, elderly, pregnant women, young children), and that only very few, usually older challenge viruses are available for use. The CHIVIM has shown that the magnitude of viral shedding correlated with illness, which in turn has aided the estimation of efficacy for current vaccines which were predictive of their field efficacy.^45^ Recent work with the CHIVIM has also identified neuraminidase inhibition titers as an independent correlate of protection.^21^ Furthermore, through work funded by the US Biomedical Advanced Research and Development Authority (BARDA), a novel vaccine approach by Vaxart was recently tested and IgA secreting cells targeting the HA were identified as a correlate of protection for this mucosally delivered vaccine (although multiple factors likely contributed to protection). Other next‐generation vaccines (eg, the Jenner Institute's MVA NP + M1, SEEK’s FLU‐v) have recently also been tested in the CHIVIM.^46^, ^47^

As reviewed during the meeting, cohort studies are also an excellent way to identify novel correlates of protection and address important questions about transmission, pathogenesis, and immunity. As an example, stalk‐reactive antibodies have recently been identified in a family cohort study in Nicaragua as an independent correlate of protection against H1N1 virus infection.^14^ Data from the same cohort also linked anti‐neuraminidase antibody titers to reduce virus shedding. The major advantages of cohort studies are that the participants acquire natural infections, they often contain individuals that belong to influenza risk groups, and they can be performed in populations that are vaccinated or non‐vaccinated, depending on the geographic location and the specific study objective.

Finally, vaccine immunogenicity and effectiveness studies are also of value to identify novel correlates of protection or to corroborate known ones. Two current trials in Hong Kong, PIVOT and RETAIN, are currently ongoing to test enhanced seasonal vaccines and repeated seasonal vaccination for the elderly. Many immunological readouts will be assessed in these studies and will be analyzed for correlation with protection. This is especially important since the HI titer of 40 is not necessarily predictive of protection in the elderly population.^48^, ^49^

## 6 CORRELATES OF PROTECTION AND ANIMAL MODELS

Immune correlates of protection can also be identified and analyzed in various animal models of influenza virus infection which include mice, hamsters, guinea pigs, ferrets, pigs, and non‐human primates (NHPs). These animal models and their respective advantages and disadvantages for correlates of protection studies were reviewed and discussed. Some of the advantages of animal models are that defined virus challenge doses can be tested, a variety of seasonal, avian, zoonotic, and pandemic viruses are available for evaluation, including viruses that may cause severe disease, and samples can be taken from multiple anatomical sites which might be hard or impossible in humans. The most common animal model is the mouse for which a large panel of immunological reagents exists. Influenza virus infection in this model is typically a lower respiratory tract infection and some mouse‐adapted seasonal (H1N1, influenza B virus), or wild type zoonotic, avian, and pandemic viruses readily induce severe disease in this model. However, seasonal H3N2 viruses typically do not replicate well in mice (sometimes not at all) and do not induce severe disease. Another advantage of mice is the existence of many different transgenic strains that can be used to find immune markers associated with protection, such as animals that lack certain Fc gamma receptors (FcγR) or have humanized FcγR, which is of high importance to study antibody effector functions. A disadvantage is the lack of the FcαR, which restricts the impact of mucosal immunity in this model. Other important disadvantages are the overall bias toward severe disease readouts and that disease in this model does not reflect influenza symptoms in humans (mice do not sneeze, cough, develop a fever (but become hypothermic), etc).

For current seasonal influenza A H3N2 viruses, which do not replicate in mice, a hamster model is now available.^50^ The small size of these animals makes them more convenient to work with and allows larger numbers per group than the ferret model (which also supports replication of H3N2 viruses). Of note, recent H3N2 isolates only amplify in the upper respiratory tract of ferrets but not in the lower respiratory tract.^51^ The ferret model has the advantage that these animals show clinical signs similar to humans including sneezing, nasal discharge, lethargy, and fever. This, together with their ability to transmit virus in aerosol‐only and contact transmission settings, makes ferret the prime animal model for influenza virus vaccine research. Disadvantages are their large size (leading to small animal numbers per group), the fact that they are often spayed/neutered (which makes studying sex differences difficult^52^), the limited availability of immunological reagents however efforts to improve availability are ongoing (see CEIRS Team Ferret Initiative^53^), their high price and maintenance cost and the poorly annotated genome (FcR, antibody germlines, etc are unknown/not well characterized).

Guinea pigs can also be a useful model to study immune markers that correlate with inhibition of transmission. Guinea pigs are small and support replication and transmission of both influenza A and influenza B viruses very well^54^, ^55^ but typically do not show any clinical signs of disease^55^ and are therefore not a good model for influenza pathogenesis. Other animal models that can be useful are pigs which are naturally infected with human and swine influenza viruses and can readily transmit these viruses within a herd. However, their large size and the need for specialized facilities are obstacles for their routine use in studies. Finally, NHPs can be used as a model for influenza infection. They are most useful for immunological studies since they show little disease when challenged with most influenza viruses, even when inoculated intratracheally. However, ethical and economic considerations do not favor the use of this model as a standard model for influenza virus vaccine development.

It should be kept in mind that immune markers identified as correlates of protection in animal models might not necessarily translate into a similar correlate of protection in humans. For example, CD8+ T‐cell immunity toward epitopes against internal conserved viral proteins in both mice and ferrets is exaggerated as compared to humans and can provide very solid protection against challenge, something not observed to the same degree in humans. Another limitation of animal models is that humans have long and complex exposure histories to influenza viruses through vaccination and/or infection, including imprinting effects.^56^, ^57^, ^58^ These complex exposure histories influence the immune responses to subsequent infection and vaccination and cannot be effectively recapitulated in animal models (pre‐exposed ferret models can be used but are very simplified compared to humans).^59^, ^60^ Furthermore, reagents and complete knowledge about immune functions (antibody subtypes/isotypes, FcRs, etc) are lacking for hamsters, ferrets, and guinea pigs. However, an advantage is that different conditions, like obesity, immune senescence, immunosuppression, pregnancy, can be tested in these models—something that cannot be done easily in humans, for example in CHIVIMs. Another interesting possibility for identifying correlates of protection in animal models is to transfer human serum into animals and determine which markers in the human serum correlate with protection of the animal from challenge.^61^

## 7 WHERE DO WE GO FROM HERE?

Two recap and discussion sessions were incorporated near the end of the meeting to synthesize the large amount of data presented over the previous two and a half days and to try to reach a consensus on where the field currently stands and what is needed to continue momentum (Table 2). Looking back at the “*Immune Correlates of Protection against Influenza”* meeting which took place in 2010 in Miami, Florida,^62^ meeting participants thought that substantial progress has been made in developing and standardizing immunological assays, and identifying novel immune markers that could serve as correlates of protection as well as *bona fide* correlates of protection. However, much work remains, as illustrated by the fact that seasonal influenza virus vaccine design and development has not been impacted in a major way by recent advances. While high dose, adjuvanted and recombinant protein based influenza virus vaccines have entered the market, they are basically similar to existing seasonal vaccines, meaning that the main focus is still to induce strong, strain specific HI/neutralizing antibody responses. Even less progress has been made with pandemic vaccines, where the concept still is to produce matched vaccines, a process that takes approximately 6 months and which unfortunately has resulted in vaccines that are available only after the first pandemic wave(s) have caused considerable morbidity and mortality—certainly an inadequate response to an emerging influenza virus. Depending on the emerging strain/subtype, stockpiled vaccines could be used, but they are limited in doses and will likely have little impact in disease burden in the general population. Furthermore, a few countries can afford this approach. The group felt that it is now time to take what has been learned in terms of immune markers and correlates of protection and start to translate this knowledge into broadly protective and universal influenza virus vaccines—while monitoring and further exploring novel correlates of protection in parallel. The participants also agreed that more work was needed on so called incremental improvements that could, in the short term, enhance the effectiveness of current seasonal vaccines. The meeting attendees were convinced that identifying influenza correlates of protection and the continued development of relevant immunological assays remain extremely important and timely and that there should be a regular series of meetings and workshops to facilitate these efforts.

**Table 2:** Areas highlighted for future work

Improved targeting of current vaccines to specific risk groups For example, LAIV to children, high dose or adjuvanted vaccines to elderly or immunosuppressed Comparative trials of licenced vaccines to guide future targeting Development and evaluation of promising next‐generation vaccine candidates in clinical trials For example, vaccines inducing NA antibodies, HA stem antibodies, protective T‐cell responses Improved use of animals models Evaluation of correlates of protection Expand immunological reagents for the ferrets (eg, CEIRS Team Ferret Initiative)^54^ Appropriate use of most relevant animal models for immunogenicity and protective efficacy based on immune mechanism of action of next‐generation influenza vaccines Human challenge model Development of new human challenge strains Standardization of the model Evaluation of broader immunity pre‐challenge to increase understanding of model Potential for use as a standardized challenge model to compare and down‐select next‐generation vaccines Cohort studies Expand cohort studies of natural infection particularly in different age and ethnic groups Standardization of immunological assays, for example, HI, VN, and stalk‐based and T‐cell assays Standardization of sample collection Harmonization of protocols Assay standardization Development and inclusion of biological standards Qualification and/or validation of assay for use in clinical trials
