# Supplementary figures and images for: Meeting report and review: Immunological assays and correlates of protection for next‐generation influenza vaccines
Source: Influenza Other Respir Viruses. 2019 Dec 13;14(2):237–43. doi: 10.1111/irv.12706 (PMC7040967; doi:10.1111/irv.12706)

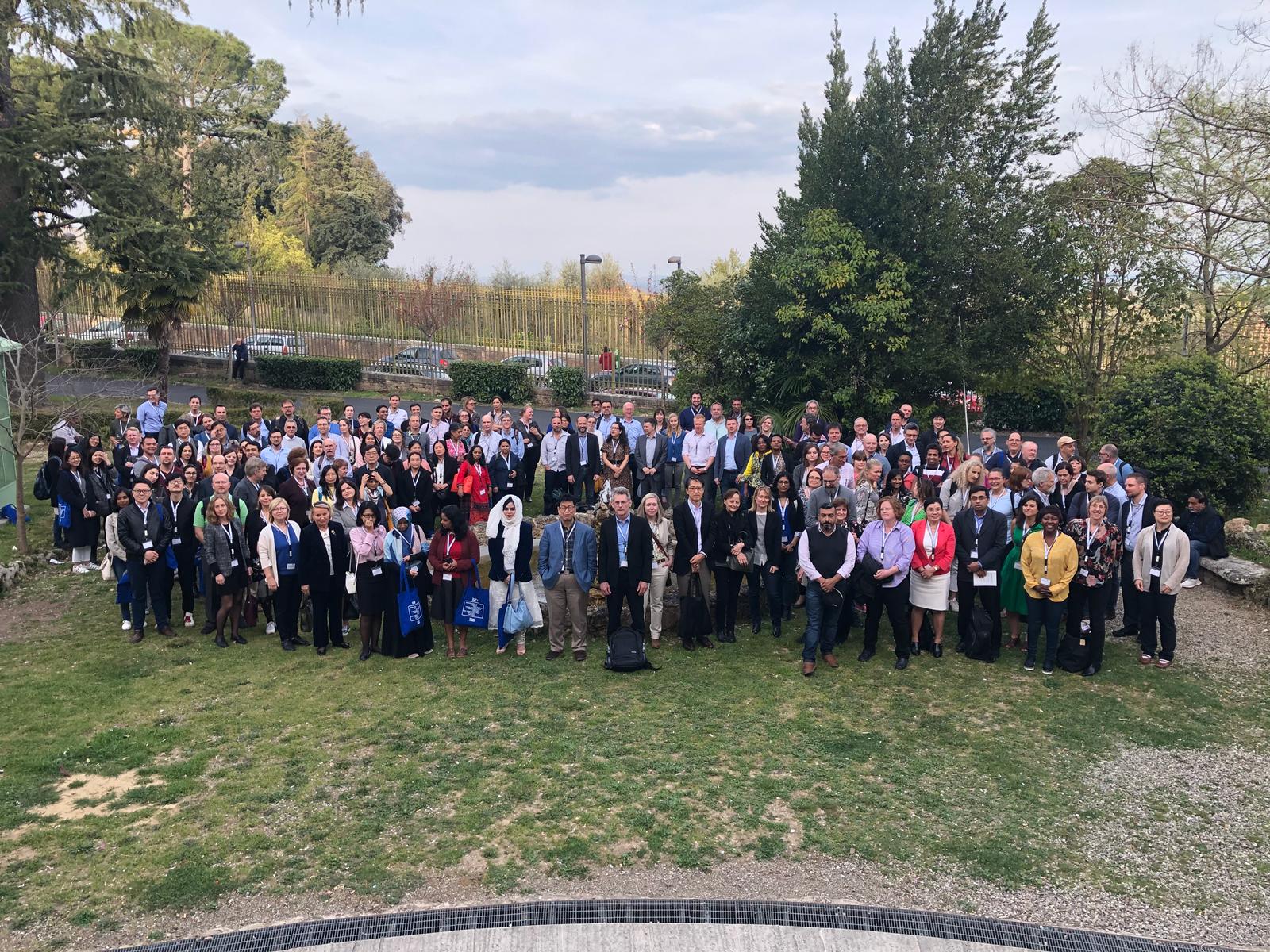

Supplement: Supplementary file 1 [file IRV-14-237-s001.jpg]
