# Supplementary material for: Meeting report and review: Immunological assays and correlates of protection for next‐generation influenza vaccines
Source: Influenza Other Respir Viruses. 2019 Dec 13;14(2):237–43. doi: 10.1111/irv.12706 (PMC7040967; doi:10.1111/irv.12706)
Supplement: Supplementary file 2 [file IRV-14-237-s002.docx]

**Immunological assays and correlates of protection for next generation influenza vaccines**

31^st^ March to 2^nd^April 2019; Siena, Italy

| **Day 1** |  |  |
| --- | --- | --- |
| **Time** | Topic | Presenter |
| **12:30 PM** | **Registration** |  |
| **2:30PM** | **Welcome and Opening Remarks** | Emanuele Montomoli  Rebecca Cox |
| **2:45 PM**  **3:25PM**  **3:55PM** | **Session A Keynote Presentations**  Influenza immunity and knowledge gaps  Overview of next generation “universal” influenza vaccines  Immune correlates of protection in humans | **Chairs: Jerry Weir, FDA, USA & Padmini Srikantiah Bill and Melinda Gates Foundation, USA**  Jackie Katz CDC  Florian Krammer Icahn School of Medicine at Mount Sinai, USA  Rebecca Cox, University of Bergen, Norway |
| **4:20 PM** | Break |  |
| **4:50 PM**  **5:20PM**  **5:45PM**  **6:10PM** | **Session B** –**Human challenge and vaccines studies**  History of human challenge systems  Evaluation of correlates of vaccine protection in the human influenza challenge model  The Human Influenza Challenge Model: Infection Rates, Virus Shedding, Symptoms and Pre-existing Humoral and Cellular Immunity  Comparative Immunogenicity of Enhanced Influenza Vaccines in Older Adults in Hong Kong:  A Randomized Controlled Trial | **Chairs: Jackie Katz CDC, USA & Stacey Schultz Cherry St Judes, USA**  Bruce Innis PATH, USA  Flora Castellino, BARDA, USA  Alex Mann, hVivo UK  Benjamin Cowling, The University of Hong Kong, Hong Kong |
| **6:30 PM** | **Opening reception** |  |

| **Day 2** |  |  |
| --- | --- | --- |
|  |  |  |
| **9:00AM**  **9:30AM**  **9:55AM**  **9:20AM** | **Session C** – **Cohorts and systems to define immune correlates of protection in humans**  Systems immunology approaches to  Cohort studies  Longitudinal cohorts to inform on influenza immunity and correlates  Risk factors and attack rates of seasonal influenza infection: results of the SHIVERS sero-epidemiologic cohort study | **Chairs Diane Post, NIH, USA & Maria Zambon, HPE, UK**  Bali Pulendran Stanford University, USA  Aubree Gordon, University of Michigan  Nancy Leung University of Hong Kong, Chin**a**  Sue Huang, Institute of Environmental Science and Research  New Zealand |
| **10:40AM** | Break |  |
| **11:10AM**  **11:40AM**  **12:10PM** | **Session D** – **Predicting vaccine performance**  Predicting vaccine performance and correlates of protection  Prime boosting studies  A case study of licensing a new influenza vaccine | **Chairs Rebecca Cox, University of Bergen & Katja Hoschler HPE, UK**  Arnold Monto  University of Michigan, USA  Kanta Subbarao  WHO Collaborating Centre for Reference & Research on Influenza, Australia  Nathalie Landry, Medicargo, Canada |
| **12:30PM** | Lunch |  |
| **2:00PM**  **2:25PM**  **2:50PM**  **3:15PM** | **Session E** –**Demonstration of protection in animal models**  From mice to Non-human primates  Demonstration of protection in ferret  Transfer models of immune effectors  Cross-reactive central and respiratory CD8 and CD4 T cells are boosted after heterosubtypic influenza infection and correlate with protection in ferrets | **Chairs Florian Krammer, Icahn School of Medicine, USA & Sarah Cobey, The University of Chicago, USA** Yoshihiro Kawaoka University of Wisconsin, USA  Stacey Schultz-Cherry  St Jude’s, USA Pierre Bruhns Institut Pasteur, France Koen van de Ven,  National Institute of Health & the Environment, Bilthoven, the Netherlands |
| **3:30PM** | Break |  |
| **4:00 PM**  **4:20PM**  **4:35PM**  **4:50PM**  **5:05PM**  **5:20PM** | **Session F** –**Immunological Assays**  Serological assays (HI virus neutralization & M2)  Stalk based antibodies  NA assays and standardisation  Local antibody assays/ novel sampling methods  Antibody Dependent Cell-mediated Cytotoxicity Assays  Functional serological assays to measure antibody responses against influenza HA globular head, HA stalk and NA | **Chairs Othmar Engelhardt NIBSC, UK & Chris Roberts, NIH, USA**    Min Levine  CDC, USA  Raffael Nachbagauer  Icahn School of Medicine at Mount Sinai, USA  Ralf Wagner,  Paul Ehrlcih Institute Germany  Katja Hoschler Public Health England, UK  Stephen Kent Doherty Institute, Australia  Nigel Temperton, University of Kent, UK |
| **5:30-7:30PM** | Poster session. |  |

| **DAY 3** |  |  |
| --- | --- | --- |
|  |  |  |
| **9:00AM**  **9:35AM**  **10:10AM**  **10:30AM**  **10:45AM** | **Session G** –**Assays for T and B cells, standardisation and harmonisation**  B cell immunity to influenza and B cellular assays  T cell immunity to influenza & T cellular assays  Standardisation of HA and NA assays (including Consise /Flucop)  Standardisation of T cellular assays for influenza  Statistical approaches | **Chairs Emanuele Montomoli University of Siena, Italy & Anke Huckriede UMCG, Netherlands**  Ali Ellebedy Washington University School of Medicine, St Louis, USA  Marios Koutsakos  Doherty Institute, Australia  Othmar Engelhardt  National Institute for Biological Standards and Control, UK  Fredrique Clement  Ghent University, Belgium  Lingyi Zheng  Sanofi Pasteur, USA |
| **11:00AM** | Break |  |
| **11:30AM** | **Session H** – **Recap of sessions B-D**  Group discussion – the way forward toward identifying correlates of protection and develop consensus on immune responses to target  Identify collaborative studies/strategies to identify correlates | **Chairs Jackie Katz, Diane Post, Rebecca Cox, Jerry Weir** |
| **12:30PM** | Lunch |  |
| **2:00PM**  **2:12PM**  **2:24PM**  **2:36PM**  **2:48PM**  **3:00PM**  **3:12PM**  **3:24PM**  **3:36PM**  **3:48PM** | **Session I Oral presentations**  Immune response after pandemic and seasonal influenza vaccination in healthcare workers  H1N1 Vaccine Effectiveness in Live Attenuated Influenza Vaccine is Driven by Viral Replicative Fitness in the Human Respiratory Tract: Selection of a new and Improved Vaccine Candidate  S-Flu: A Potential Universal Influenza Vaccine  Assessment of the correlation between VaxArray alternative potency assays for hemagglutinin and neuraminidase and immunogenicity in mice  The Many Confounding Variables with Hemagglutination Inhibition Assays  Determining the correlation between the anti-hemagglutinin stalk activity of serum samples in an ADCC Reporter Bioassay versus a flow cytometry-based CD107a degranulation assay  Influenza virus infection as well as immunization induces high titer cross-reactive haemagglutinin-specific antibody-dependent phagocytosis (ADP) and monocyte infection-enhancing responses in macaques  Generation of an international standard serum to measure influenza virus hemagglutinin stalk-reactive antibodies  Contributions of hemagglutinin- and neuraminidase-specific antibodies for protection against influenza during a 2016 influenza A/H3N2 outbreak in the military  Reduced antibody function correlates with influenza infection in a household model of transmission | **Chairs Arnold Monto, University of Michigan, USA & Kanta Subbaroa, WHO Australia**  Mai Chi Trieu,  University of Bergen, Norway  Oliver Dibben, Astrazeneca/MedImmune, UK  Tiong Kit Tan,  University of Oxford, UK  Rose Nash,  InDevR, Inc., USA  Ewan P. Plant,  Food and Drug Administration, USA  Veronika Chromikova,  Icahn School of Medicine, USA  Edmond Remarque,  BPRC, Netherlands  Juan Manuel Carreno Quiroz,  Icahn School of Medicine, USA  Carol Weiss,  US FDA, Center for Biologics Evaluation and Research, USA  Sophie Valkenburg, The University of Hong Kong, Hong Kong |
| **4:00PM** | Break |  |
| **4:30PM** | **Session J** -**Recap of sessions E-G**    Group discussion – the way forward in developing and standardizing assays needed for measuring the relevant immune responses of next generation vaccines.  What are relevant immune responses?  Best assays available  Approaches to standardisation and harmonisation | **Chairs Florian Krammer**  **Othmar Engelhardt, , Emanuele Montomoli, Chris Roberts** |
| **5:30PM** | Close of meeting and closing remarks |  |
